# Differences between evidence-based recommendations and actual clinical practice regarding tocolysis: a prospective multicenter registry study

**DOI:** 10.1186/s12884-018-2078-5

**Published:** 2018-11-16

**Authors:** Emina Nazifovic, Heinrich Husslein, Ioana Lakovschek, Florian Heinzl, Elisabeth Wenzel-Schwarz, Philipp Klaritsch, Ekrem Kilic, Sarah Hoesel, Rudolf Bind, Magdalena Pabinger, Harald Zeisler, Lorenz Kuessel

**Affiliations:** 1 Department of Gynecology and Obstetrics, Semmelweis Frauenklinik, Bastiengasse 36-38, A-, 1180 Vienna, Austria; 2 Department of Gynecology and Obstetrics, Medical University of Vienna, Waehringer Guertel 18-20, A-, 1090 Vienna, Austria; 3 Department of Obstetrics and Gynecology, Medical University of Graz, Auenbruggerplatz 14, A – 8036 Graz, Graz, Austria; 4 Department of Gynecology and Obstetrics, St. Josef Krankenhaus, Auhofstrasse 189, A-, 1130 Vienna, Austria; 5 Department of Gynecology and Obstetrics, Medical University of Krems, University Hospital of Tulln, Alter Ziegelweg 10, A-3430 Tulln, Austria; 6 Department of Gynecology and Obstetrics, Klinikum Klagenfurt am Wörthersee, Feschnigstraße 11, A-, 9020 Klagenfurt am Wörthersee, Austria; 7 Department of Gynecology and Obstetrics, Landesklinikum Zwettl, Probstei 5, A-, 3910 Zwettl, Austria; 8 Department of Gynecology and Obstetrics, Krankenanstalt Rudolfstiftung, Juchgasse 25, A-, 1030 Vienna, Austria

## Abstract

**Background:**

International guidelines recommend that tocolytic therapy be restricted to a single 48-h application. However, multiple cycles of tocolytic therapy and maintenance therapy that exceeds 48 h appear to play a role in daily clinical practice. We aimed to evaluate current trends in clinical practice with respect to treatment with tocolytic agents and to identify differences between evidence-based recommendations and daily clinical practice in Austria.

**Methods:**

A prospective multicenter registry study was conducted from October 2013 through April 2015 in ten obstetrical departments in Austria. Women ≥18 years of age who received tocolytic therapy following a diagnosis of threatened preterm birth were included, and details were obtained regarding clinical characteristics, tocolytic therapy, and pregnancy outcome.

**Results:**

A total of 309 women were included. We observed a median of 2 cycles of tocolytic therapy per patient (IQR 1–3) with a median duration of 2 days per cycle (IQR 2–5). Repeat tocolysis was administered in 41.7% of women, resulting in up to six tocolysis cycles; moreover, 40.8% of the first tocolysis cycles were maintenance tocolysis (i.e., longer than 48 h). Only 25.6% of women received one single 48-h tocolysis cycle in which they received antenatal corticosteroids for fetal lung maturation in accordance evidence-based recommendations.

**Conclusions:**

Here, we report a clear disparity between evidence-based recommendations and daily practice with respect to tocolysis. We believe that the general practice of prescribing tocolytic therapy must be revisited. Furthermore, our findings highlight the need for contemporary studies designed to investigate the effectiveness of performing maintenance and/or repetitive tocolysis treatment.

**Electronic supplementary material:**

The online version of this article (10.1186/s12884-018-2078-5) contains supplementary material, which is available to authorized users.

## Introduction

Gestational age and birth weight are major contributors to neonatal morbidity and mortality [1, 2]. Preterm birth (PTB), which is defined as birth prior to 37 full weeks of gestation, represents one of the most significant challenges in modern obstetrics and perinatal medicine. In Europe, approximately 7% of all pregnancies end in PTB, and several studies revealed an increase in the prevalence of PTB over the past 20 years [3, 4]. Moreover, PTB-associated neonatal and long-term morbidity are associated with significant socioeconomic costs [5].

Although the precise mechanisms underlying PTB are not fully understood, preterm labor (PTL) is considered the common final step in a variety of pathophysiological processes with multifactorial causes, which are often difficult to treat individually [6–8]; additionally, PTL is often difficult to predict and identify [6, 8]. Therefore, the use of tocolytic therapy to prevent PTL is an attractive option in efforts aiming to prevent PTB. However, the efficacy of tocolysis in terms of reducing infant morbidity or mortality, as well as long-term outcome, is still a matter of debate. Although tocolytic agents have been shown to slightly prolong pregnancy in the setting of PTL, evidence regarding a general improvement in neonatal outcome is currently lacking [5, 9, 10]; indeed, a positive effect of tocolysis on neonatal outcome is evident only when PTB is delayed long enough to allow the use of antenatal corticosteroids for promoting fetal lung maturation [11].

Since maintenance therapy with tocolytic drugs has not been shown to effectively prevent PTB or improve neonatal outcome, its use is not recommended [5, 12, 13]. Therefore, both national and international guidelines recommend limiting tocolytic therapy to a single treatment cycle that does not exceed 48 h [5, 14–18].

Despite these recommendations, and in contrast with these guidelines, multiple cycles of tocolytic therapy and maintenance therapy that exceeds 48 h appear to be relatively common in daily clinical practice [19]. This notion is supported by personal communications with clinicians and scientists in German-speaking countries.

The purpose of this study was to *i*) examine the current state of clinical practice in Austria with respect to the use of tocolytic therapy, and *ii*) evaluate the differences between evidence-based recommendations and daily clinical practice.

## Materials and methods

Pregnant women who were admitted to one of ten participating hospitals in Austria from October 2013 through April 2015 with a diagnosis of threatened PTB were invited to participate in the prospective multicenter registry study FRÜSGO. The FRÜSGO study was designed to survey/observe the practice of applying tocolytic agents for managing PTB in Austria and was developed and supervised by the Fetomaternal Research Group, which is affiliated with the Austrian Society of Obstetrics and Gynecology. During initiation of the FRÜSGO study, tocolytic doses were reported to be identical and administration procedures very similar by all participating study sites.

Participation in the study had no effect on local standards, definitions, or guidelines, nor did it affect either the indication for tocolysis or the type or duration of tocolytic therapy. In our study, we enrolled women ≥18 years of age who received tocolytic therapy for the first time in the current pregnancy. Ten centers participated in the study; five perinatal referral centers; two centers who averaged more than 1000 deliveries per year, and three centers who averaged fewer than 1000 deliveries per year. All patients provided written informed consent prior to participation. Upon enrollment, each patient’s epidemiological data and clinical characteristics were obtained. For each tocolysis cycle, we documented the diagnosis, physical examination results, the indication for tocolytic therapy, the type and duration of tocolytic therapy, and whether antenatal corticosteroids were administered concurrently. During and after each tocolysis cycle any adverse effect was noted by the nursing staff. Additionally, patients were asked to report any subjective adverse effects that occurred. Subsequently, parturition details and neonatal outcome were obtained. Medical data were documented in the form of an electronic case report using an online database available at www.scicomed.net. Out of a total of 340 women who were included to the present study, 31 (9%) women had to be excluded from analysis due to insufficient data quality regarding type and duration of tocolysis.

A single cycle of tocolysis therapy was defined as the time between the beginning and end of a treatment round with a tocolytic drug. A 48-h tocolysis cycle was defined as a cycle that lasted 46–50 h; a cycle that lasted longer was defined as maintenance tocolysis. If a patient who had completed at least one prior cycle of tocolysis subsequently received any further tocolytic therapy (e.g., readmission to the hospital due to PTL), this was defined as repeat tocolysis.

To identify differences between evidence-based recommendations and clinical practice, we compared women who received tocolytic therapy as recommended by the guidelines with women who received tocolytic therapy in conflict with these recommendations. The use of tocolytic therapy in a patient was considered evidence-based (EVB group) if a single tocolytic cycle was administrated after 23 + 0 weeks of gestation using a single tocolytic agent. Any repetition or other form of tocolytic therapy was considered to be non-evidence–based (the non-EVB group). For comparisons between the EVB and non-EVB group, we excluded all women who received a cycle of tocolytic therapy that was shorter than 48 h as a result of preterm birth.

The statistical software package R (version 3.4.0) was used for data analyses. For descriptive statistics, linear variables are reported as the mean ± the standard deviation, ordinal variables are reported as the median and the interquartile range (IQR), and categorical variables are reported as the absolute or relative frequency. Patient characteristics were compared between groups using Wilcoxon rank sum test (for ranked variables) or Pearson’s Chi-squared test with Yates’ continuity correction (for nominal variables). All analyses were two-sided. Because we tested the EVB group against three different groups, we chose to employ Bonferroni correction. A *p*-value of at most 0.05/3 ~ 0.0166 was considered statistically significant.

## Results

### Patient characteristics and tocolysis regimens

A total of 309 women were included in our analysis; the patient characteristics are summarized in Table 1. Among these 309 women, a total of 509 tocolysis cycles were administrated; with a median (IQR) of 2 (2–5) cycles. The observed clinical findings and indications for each respective tocolysis cycle are summarized in Table 2. A total of 180 women (58.3%) received a single treatment cycle and 129 women (41.7%) received multiple treatment cycles. Among the 180 women who received only one cycle, 18 (10%) delivered within 48 h, 96 (53.3%) received a 48-h treatment cycle, and the remaining 66 (36.7%) received maintenance therapy. Among the 129 women who received multiple treatment cycles, 82, 30, 12, 3, and 2 patients received two, three, four, five, or six cycles, respectively.

**Table 1:** Patient characteristics, pregnancy outcome parameters, and differences among women who received tocolytic therapy as recommended in the guidelines (EVB) and women who received tocolytic therapy tocolysis in contrast with these recommendations (nonEVB)

	Total	EVB	nonEVB	p EVB vs nonEVB
Women (n)	309	93	198	
Characteristics				
Maternal Age	30.78 ± 5.8	30.35 ± 6.0	30.93 ± 5.6	0.699^a^
Maternal BMI	23.25 ± 4.7	23.25 ± 4.6	23.17 ± 4.8	0.676^a^
Smoking in pregnancy	37 (12.0%)	14 (15.1%)	21 (10.6%)	0.371 ^b^
ART	58 (18.7%)	12 (12.9%)	34 (17.2%)	0.470^b^
Parity	0.64 ± 0.9	0.72 ± 1.0	0.62 ± 0.9	0.372 ^a^
Multiple pregancy	85 (27.5%)	18 (19.4%)	57 (28.8%)	0.116^b^
GA at 1st TL (weeks)	28 (25–31)	30 (27–32)	27 (24–30)	< 0.001^a^
Children (n)	395	111	265	
Pregnancy Outcome				
GA at delivery (weeks)	35 (31–38)	38 (35–39)	34 (30–38)	< 0.001^a^
< 28 + 0	31 (10.0%)	4 (4.3%)	23 (11.6%)	< 0.001^a^
28 + 0–33 + 6	83 (26.9%)	10 (10.8%)	64 (32.3%)	
> 34 + 0	195 (63.1%)	79 (84.9%)	111 (56.1%)	
Birth weight (g)				
< 1000	33 (8.4%)	2 (1.8%)	29 (11.1%)	< 0.001^a^
1000–2000	117 (29.9%)	20 (18.2%)	82 (31.4%)	
> 2000	242 (61.7%)	88 (80%)	150 (57.5%)	
UApH	7.3 ± 0.2	7.29 ± 0.1	7.31 ± 0.2	0.562^a^
Transfer ad NICU	203 (51.4%)	35 (31.5%)	153 (57.8%)	< 0.001^b^

Notes: Categorical data are presented as the frequency and percentage (rounded). Continuous variables are expressed as the mean ± SD, ordinal variables as the median (IQR). Women who delivered within 48 h after beginning of tocolytic treatment (*n* = 18) were excluded from EVB vs nonEVB analysis

*BMI* body mass index, *PTB* preterm birth, *ART* assisted reproductive technology, *GA* gestational age, *TL* treatment with tocolytics, *UAPH* PH value of the umbilical artery, *NICU* neonatal intensive care unit, *EVB* evidence based, *non EVB* not evidence based – as defined above

^a^Wilcoxon rank sum test

^b^Pearson’s Chi-squared test with Yates’ continuity correction

**Table 2:** Clinical findings and documented indications at tocolysis cycle 1, 2, and 3

Tocolysis cycle		Total	1st	2nd	3rd
Patients (n)		309/509 cycles	309	129	47
Clinical findings					
Preterm labor		280 (55.0%)	148 (47.9%)	83 (64.3%)	32 (68.1%)
Isolated CXI		146 (28.7%)	98 (31.7%)	92 (71.3%)	37 (78.7%)
PROM		38 (7.5%)	29 (9.4%)	5 (3.9%)	2 (4.3%)
Genital Infection		20 (3.9%)	13 (4.2%)	4 (3.1%)	3 (6.4%)
CX < 25 mm		379 (74.5%)	227 (73.5%)	103 (79.8%)	30 (63.8%)
Cervical length		34.6 ± 51.9	36.06 ± 56.0	33.41 ± 48.2	31.43 ± 42.21
Multiple prenancy		148 (29.1%)	78 (25.2%)	42 (32.6%)	21 (44.7%)
Indication for tocolysis					
Lung maturation		289 (56.8%)	234 (75.7%)	40 (31.0%)	8 (17.0%)
Prolongation of pregnancy		218 (42.8%)	73 (23.6%)	89 (69.0%)	39 (83.0%)
Transfer to center		2 (0.4%)	2 (0.7%)	0 (0.0%)	0 (0.0%)
GA at beginning (weeks)		28.32 ± 3.3	27.68 ± 3.5	29.09 ± 2.9	29.32 ± 2.7
Duration of tocolysis (days)		4.8 ± 6.3	4.78 ± 6.8	4.61 ± 5.7	4.63 ± 4.7
Type	Atosiban	439 (86.2%)	263 (85.1%)	115 (89.1%)	39 (83.0%)
	Hexoprenaline	43 (8.5%)	30 (9.7%)	8 (6.2%)	5 (10.6%)
	Nifedipine	2 (0.4%)	0 (0.0%)	1 (0.8%)	0 (0.0%)
	Switch of substance/double medication	25 (4.9%)	16 (5.2%)	5 (3.9%)	3 (6.4%)

*CXI* cervical insufficiency, *PROM* preterm rupture of membranes, *CX* cervical length, *GA* gestational age

**Fig. 1:**
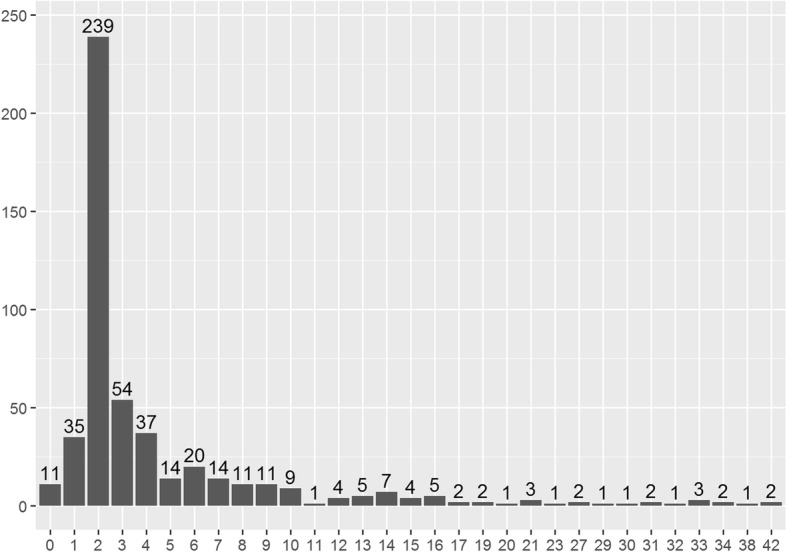
Distribution of duration of tocolytic cycles. Notes: Respective duration of 509 cycles of treatment with tocolytics administered in 309 women. Days, duration of tocolysis cycle in days. x-axis: duration (days) y-axis: number of women (n)

The median duration of the tocolysis cycles was 2 (2–5) days. Although more than three-quarters of the tocolysis cycles (*N* = 239, 77.3%) had a length of 48 h, we observed wide heterogeneity with respect to the duration of maintenance tocolysis regimens (Fig. 1). The maximum length of tocolytic treatment among our patients was 48 days.

**Fig. 2:**
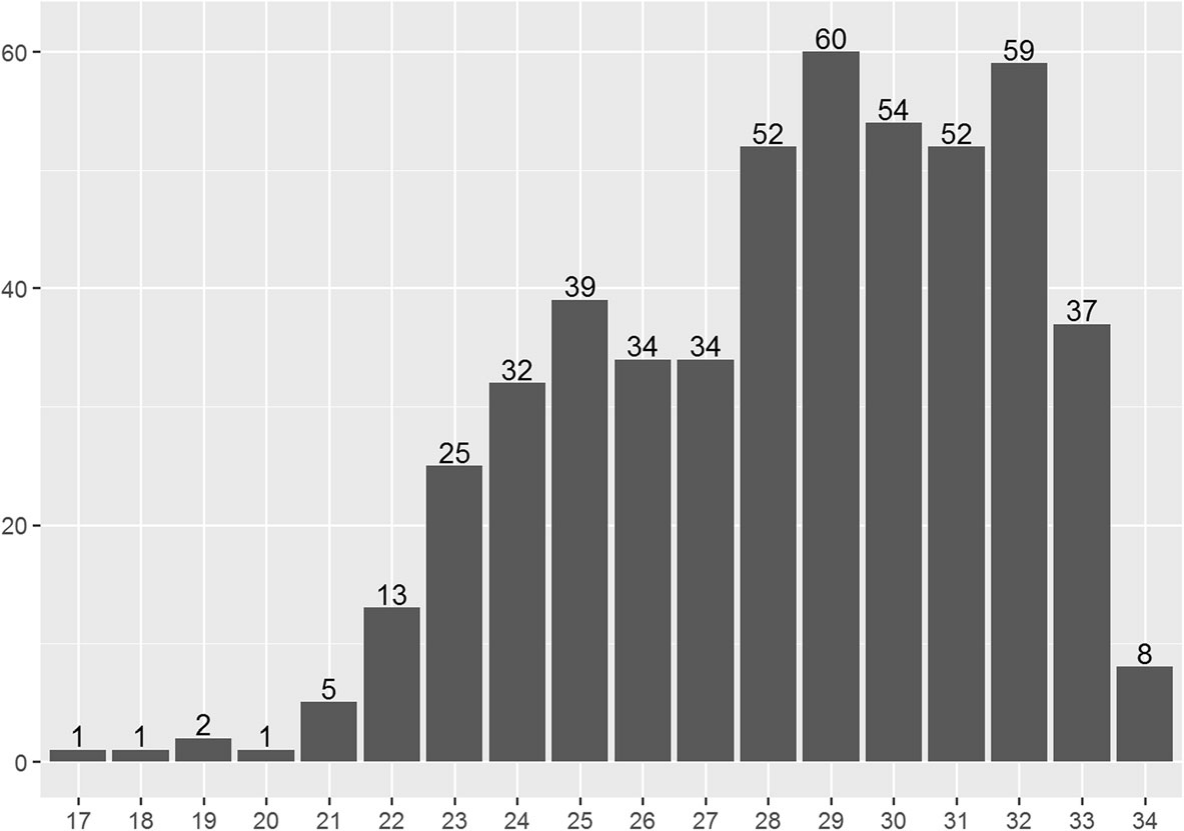
Distribution of gestational age at tocolysis over the weeks of gestation. Notes: Respective gestational age at the initiation of 509 cycles of treatment with tocolytics administered in 309 women. x-axis: gestational age (weeks) y-axis: number of cycles (n)

The number of tocolysis cycles is plotted against gestational age (GA) in Fig. 2. The median GA at the beginning of tocolysis was 28 (25–31) weeks. In 4.5% of all cases (23 cycles), tocolysis was initiated prior to 23 + 0 weeks of gestation (i.e., the gestational threshold for neonatal viability).

The most commonly used tocolytic agent was the oxytocin receptor antagonist atosiban (*N* = 439 treatment cycles, 86.2%), followed by the beta-agonist hexoprenaline (N = 43, 8.5%), and the calcium channel blocker nifedipine (N = 2, 0.4%). In 25 treatment cycles (4.9%), the tocolytic agent was switched during the cycle, or a combination of both hexoprenaline and atosiban was administered simultaneously.

In total, four adverse effects in response to tocolytic therapy were documented. Specifically, flush-like symptoms and hypotension with vertigo were each reported once, and headache was reported twice; in all four cases, patients were receiving atosiban.

### Clinical findings and indications for tocolysis

The documented clinical findings for tocolysis, as well as the indications, are summarized in Table 2. The most common clinical finding was preterm labor (PTL, 55% of cycles), followed by isolated cervical insufficiency (28.7% of cycles). Administration of antenatal corticosteroids was the reported indication for the first cycle of tocolytic therapy in 75.7% of patients. In cases of repeat treatment cycles, the most common indication for tocolysis was prolongation of pregnancy (see Table 2).

#### Group comparisons

Next, we compared the EVB group with the non-EVB group. Women who delivered within 48 h after beginning of tocolytic treatment (*n* = 18) were excluded from this analysis. Among the 96 patients who received a single 48-h cycle, one received simultaneous double tocolysis, and two patients received tocolytic treatment before 23 + 0 weeks of gestation. These women (*n* = 3) were included to the non-EVB group in addition to women who received multiple treatment cycles (*n* = 129) and women who received one cycle the lasted > 48 h (*n* = 66). A total of 93 women received a single tocolysis cycle as recommended and were included in the EVB group; 79 of these 93 patients (84.9, 25.6% of total sample size) received antenatal corticosteroids, 14 (15.1%) did not receive antenatal steroids.

Table 1 summarizes the differences between the EVB group and the non-EVB group. We found no significant differences between groups with respect to general clinical characteristics. However, patients in the non-EVB group received their first tocolytic treatment at a significantly earlier gestational age compared to patients in the EVB group (median of 27 (24–30) weeks vs. 30 (27–32) weeks, respectively; *p* < 0.001). With respect to neonatal outcomes, we found that women in the non-EVB group delivered babies (i) at earlier GA (ii) with lower weight, who (iii) had to be transferred to a neonatal intensive care unit more often when compared to women in the EVB group (all p < 0.001). Subgroup characteristics of the women who received multiple treatment cycles (repetitive group), and the women who received one cycle of tocolysis the lasted > 48 h (maintenance group) are provided in Additional file 1: Table S1.

## Discussion

The Austrian guidelines for managing preterm labor call for tocolytic treatment for the short-term prolongation of pregnancy (up to 48 h) in order to administer a single course of antenatal corticosteroids. Consistent with leading international guidelines, both maintenance therapy and repeat tocolytic therapy are not recommended [17]. Here, we evaluated the current clinical practice of tocolytic treatment in Austria. Our analysis revealed that the majority of participating women received tocolytic therapy in a regimen that is in clear contrast with the recommendations. Both maintenance therapy and repeat tocolytic therapy were common in our study. Strikingly, only one quarter of the patients received a single 48-h tocolysis cycle in which they received antenatal corticosteroids for fetal lung maturation in accordance with the recommended treatment; Even in the EVB group 15.1% of women did not receive antenatal steroids, representing another area for improvement. Although we found no significant differences between the EVB and non-EVB group with respect to general clinical characteristics, the women in the non-EVB group received their initial tocolytic treatment at an earlier gestational age than the women in the EVB group (median 27 vs. 30 weeks, respectively).

It is important to note that our study was not designed to determine whether repeat cycles and/or maintenance tocolytic treatment can improve neonatal outcome. Moreover, this study had a number of limitations that warrant discussion. First, the relatively wide use of atosiban among our patients might not reflect international usage, as atosiban is not well established in other countries. However, unlike other tocolytic agents (e.g., nifedipine), atosiban is licensed for use in the European Union for treating patients who are at risk for preterm birth, which may explain the wide use of atosiban in our population. Second, the relatively limited sample size precluded statistical analyses based on the type or size of the participating centers. However, it should be noted that FRÜSGO was designed to evaluate patterns in clinical practice regarding tocolytic treatment without evaluating or comparing the management of individual institutions or clinicians.

A strength of our study was its prospective design, which resulted in a well-characterized sample that likely represents current clinical practice in Austria. This design enabled us to identify a clear gap between evidence-based recommendations and clinical practice with respect to tocolytic treatment.

Published reports suggest that prolonging pregnancy with repeat tocolytic cycles or maintenance tocolytic therapy is ineffective and does not improve neonatal outcome [5]. In addition, current literature does not support the notion that tocolytic treatment generally has any direct benefit with respect to neonatal outcome. Thus, the decision to use repeat cycles and/or maintenance treatment with tocolytic agents is not based on evidence-based clinical benefits, but rather driven by local customs and individual experience (i.e., the clinician’s “gut feeling”). Additionally, a clinician’s decision to maintain tocolysis or repeatedly administer tocolytic agents may be affected by the patient’s own fear of ending treatment, which may cause the patient to pressure the clinician to maintain and/or repeat tocolytic therapy. This is in line with publications proposing an increasing awareness of the need to tailor clinical management to specific patient needs and placing the patient into the role of a shared decision maker with their healthcare professionals [20]. Moreover, many clinicians apparently consider tocolytic treatment as a low risk intervention; a notion that is supported by a survey among clinicians in the United States [19]. It revealed that many clinicians believe that tocolysis has other benefits in addition to facilitating the use of antenatal corticosteroids and that they would be open to continuing tocolytic treatment if requested by the patient [19]. In the current study this effect may be intensified given that atosiban is the first tocolytic of choice in Austria and promises a favorable risk/benefit profile due to a low incidence of side effects reported in literature; In this respect, it is worth noting that the incidence of side effects was low in our study (only 1.2% of cases), and no severe maternal or fetal adverse events were documented, which is consistent with previous studies [21].

Many of the studies on which current recommendations are based were conducted decades ago, had limited sample sizes and may therefore be outdated [22]. For example, in our patient population the median gestational age at the start of tocolysis was 28 weeks; in contrast, the most recent meta-analyses reviewing the use of betamimetics to inhibit PTL reported that the gestational age was usually ≥32 weeks [23]. The most recent Cochrane review on oxytocin receptor antagonists for inhibiting preterm labour included only 44 women receiving atosiban in due to PTB before 28 weeks´ of gestation [10]. With continuous improvements in neonatal care, which has increased the survival rate of even extremely preterm infants, clinicians nowadays more commonly face clinical situations of PTL at earlier gestational age. Therefore, the question has to be raised, whether the perinatal challenges that clinicians face in daily practice are adequately covered by current practice guidelines.

This notion is supported by our findings that women in the non-EVB group received their first tocolytic treatment at earlier gestational age than the EVB group, and that cervical length decreased with each successive cycle of tocolysis. In addition we found that women in the non-EVB group gave birth at earlier gestational age than women in the EVB group. Taken together, these findings suggest that the women in the non-EVB group presented with a higher-risk clinical condition, and given the severity of their patients’ symptoms, that clinicians may be more open to treating their patients with repetitive tocolytic treatment or maintenance therapy, particularly given the favorable risk profile associated with atosiban.

Although one can hypothesize that the non-evidence based trends described above are likely present in numerous other institutions throughout the industrialized world, it is important to note once again, that the findings of our study were derived from data collected in one single country. Therefore we recommend validating the findings of our study in larger, international investigations. Confirmation of a widespread need to improve clinical practice regarding tocolytic therapy could drive a push for further effectiveness and safety studies and ultimately to update guidelines to inform evidence-based obstetric practice.

## Conclusion

The use of tocolytic agents with the intention to prolong pregnancy has become deeply ingrained in obstetric practice. Here, we report a clear disparity between evidence-based recommendations and daily practice with respect to tocolysis. We believe that the general practice of prescribing tocolytic therapy must be revisited. Furthermore, our findings highlight the need for contemporary studies designed to investigate the use and potential harm of performing maintenance and/or repetitive tocolysis treatment.

## Additional file

Additional file 1: **Table S1.** Characteristics of the patients who received repeat tocolysis, and the patients who received maintenance therapy. Notes: Categorical data are presented as the frequency and percentage (rounded). Continuous variables are expressed as the mean ± SD, ordinal variables as the median (IQR). Women who delivered within 48 h after beginning of tocolytic treatment (*n* = 18) were excluded from EVB vs nonEVB analysis. BMI, body mass index; PTB, preterm birth; ART, assisted reproductive technology; GA, gestational age; TL, treatment with tocolytics; UAPH, PH value of the umbilical artery; NICU, neonatal intensive care unit; EVB, evidence based; non EVB, not evidence based – as defined above. ^a^Wilcoxon rank sum test; ^b^Pearson’s Chi-squared test with Yates’ continuity correction. (DOCX 18 kb)

## Abbreviations

BMI: Body mass index
CX: Cervical length
CXI: Cervical insufficiency
EVB: Evidence based
GA: Gestational age
NICU: Neonatal intensive care unit
nonEVB: Not evidence based
PROM: Preterm rupture of membranes
PTB: Preterm birth
PTL: Preterm labour
SD: Standard deviation
TL: Treatment with tocolysis; UApH, Umbilical artery pH value

## References

[1] American College of Obstetricians Gynecologists, Committee on Practice Bulletins-Obstetrics (2012). Practice bulletin no. 130: prediction and prevention of preterm birth. Obstet Gynecol.

[2] Stein W, Jahns B, Hawighorst T, Emons G (2009). Long-term tococlysis with beta-2-mimetics-a retrospective analysis from one centre. Z Geburtshilfe Neonatol.

[3] Scheuchenegger A, Lechner E, Wiesinger-Eidenberger G, Weissensteiner M, Wagner O, Schimetta W, Resch B (2014). Short-term morbidities in moderate and late preterm infants. Klin Padiatr.

[4] Butler AS, Behrman RE. Preterm birth: causes, consequences, and prevention. Washington: National Academies Press; 2007. 20669423

[5] American College of Obstetricians Gynecologists, Committee on Practice Bulletins-Obstetrics (2016). Practice bulletin no. 171: Management of Preterm Labor. Obstet Gynecol.

[6] Lockwood CJ, Kuczynski E (2001). Risk stratification and pathological mechanisms in preterm delivery. Paediatr Perinat Epidemiol.

[7] Navathe R, Berghella V (2016). Tocolysis for acute preterm labor: where have we been, where are we now, and where are we going?. Am J Perinatol.

[8] Romero R, Dey SK, Fisher SJ (2014). Preterm labor: one syndrome many causes. Science.

[9] Elliott JP, Morrison JC (2013). The evidence regarding maintenance tocolysis. Obstet Gynecol Int.

[10] Flenady V, Reinebrant HE, Liley HG, Tambimuttu EG, Papatsonis DN (2014). Oxytocin receptor antagonists for inhibiting preterm labour. Cochrane Database Syst Rev.

[11] Haas DM, Caldwell DM, Kirkpatrick P, McIntosh JJ, Welton NJ (2012). Tocolytic therapy for preterm delivery: systematic review and network meta-analysis. BMJ.

[12] Roberts D, Dalziel S. Antenatal corticosteroids for accelerating fetal lung maturation for women at risk of preterm birth. Cochrane Database Syst Rev. 2006;3:CD004454. 10.1002/14651858.CD004454.pub2 16856047

[13] Papatsonis DN, Flenady V, Liley HG (2013). Maintenance therapy with oxytocin antagonists for inhibiting preterm birth after threatened preterm labour. Cochrane Database Syst Rev.

[14] Lamont RF (2003). International preterm labour C: evidence-based labour ward guidelines for the diagnosis, management and treatment of spontaneous preterm labour. J Obstet Gynaecol.

[15] Sentilhes L, Senat MV, Ancel PY, Azria E, Benoist G, Blanc J, Brabant G, Bretelle F, Brun S, Doret M (2016). Prevention of spontaneous preterm birth: guidelines for clinical practice from the French College of Gynaecologists and Obstetricians (CNGOF). Eur J Obstet Gynecol Reprod Biol.

[16] Beinder E, Dudenhausen J, Feige A, Hackelöhr B, Hecher K, Rath W. Medikamentöse Wehenhemmung bei drohender Frühgeburt. AWMF Deutsche Gesellschaft für Gynäkologie und Geburtshilfe. 2006;015–25 German.

[17] Mad P, Geiger-Gritsch S, Mittermayr T, Wild C. Medikamentöse Wehenhemmung bei drohender Frühgeburt - Systematischer Review zu Leitlinien, Wirksamkeit und Gesundheitsökonomischen Evaluationen der Tokolyse. Wien: Ludwig Boltzmann Institut, HTA-Projektbericht #030; 2009.

[18] RCOG. Tocolysis for women in preterm labour. London: Royal College of Obstetricians and Gynaecologists; 2011. p. 1–13. Green-top Guideline No 1b.

[19] Fox NS, Gelber SE, Kalish RB, Chasen ST (2008). Contemporary practice patterns and beliefs regarding tocolysis among u.s. maternal-fetal medicine specialists. Obstet Gynecol.

[20] Chervenak FA, McCullough LB (2017). The unlimited-rights model of obstetric ethics threatens professionalism. BJOG.

[21] de Heus R, Mol BW, Erwich JJ, van Geijn HP, Gyselaers WJ, Hanssens M, Harmark L, van Holsbeke CD, Duvekot JJ, Schobben FF (2009). Adverse drug reactions to tocolytic treatment for preterm labour: prospective cohort study. BMJ.

[22] Walker KF, Thornton JG (2016). Tocolysis and preterm labour. Lancet.

[23] Neilson JP, West HM, Dowswell T (2014). Betamimetics for inhibiting preterm labour. Cochrane Database Syst Rev.

