# Supplementary material for: Differences between evidence-based recommendations and actual clinical practice regarding tocolysis: a prospective multicenter registry study
Source: BMC Pregnancy Childbirth. 2018 Nov 16;18:446. doi: 10.1186/s12884-018-2078-5 (PMC6240217; doi:10.1186/s12884-018-2078-5)
Supplement: Supplementary file 1 — Table S1. Characteristics of the patients who received repeat tocolysis, and the patients who received maintenance therapy. Notes: Categorical data are presented as the frequency and percentage (rounded). Continuous variables are expressed as the mean ± SD, ordinal variables as the median (IQR). Women who delivered within 48 h after beginning of tocolytic treatment (n = 18) were excluded from EVB vs nonEVB analysis. BMI, body mass index; PTB, preterm birth; ART, assisted reproductive technology; GA, gestational age; TL, treatment with tocolytics; UAPH, PH value of the umbilical artery; NICU, neonatal intensive care unit; EVB, evidence based; non EVB, not evidence based – as defined above. aWilcoxon rank sum test; bPearson’s Chi-squared test with Yates’ continuity correction. (DOCX 18 kb) [file 12884_2018_2078_MOESM1_ESM.docx]

**Table S1**

|  | **EVB (1)** | **Repeat (2)** | **Maintenance (3)** | **p 1 vs 2** | **p 1 vs 3** | **p 2 vs 3** |
| --- | --- | --- | --- | --- | --- | --- |
| **Women (n)** | 93 | 129 | 66 |  |  |  |
| **Characteristics** |  |  |  |  |  |  |
| Maternal Age | 30.35±6.0 | 31.19±5.6 | 30.36±5.5 | 0.591^a^ | 0.967^a^ | 0.6192^a^ |
| Maternal BMI | 23.25 ± 4.6 | 23.16 ± 4.8 | 23.3 ± 5.1 | 0.767^a^ | 0.738^a^ | 0.8633^a^ |
| Smoking in pregnancy | 14 (15.1%) | 15 (11.6%) | 5 (7.6%) | 0.585 ^b^ | 0.236 ^b^ | 0.5266^b^ |
| ART | 12 (12.9%) | 24 (18.6%) | 9 (13.6%) | 0.358^b^ | 1^b^ | 0.5005^b^ |
| Parity | 0/1 | 0/1 | 0/1 | 0.608 ^a^ | 0.226 ^a^ | 0.5715^a^ |
| Multiple pregancy | 18 (19.4%) | 42 (32.6%) | 15 (22.7%) | 0.042^b^ | 0.750^b^ | 0.207^a^ |
| GA at 1st TL (weeks) | 30/5 | 27/5 | 28/5 | <0.001^a^ | <0.001^a^ | 0.1184^a^ |
| **Children (n)** | 111 | 170 | 83 |  |  |  |
| **Pregnancy Outcome** | |  |  |  |  |  |
| GA at delivery (weeks) | 38/4 | 35/7 | 34/9 | <0.001^a^ | <0.001^a^ | 0.3386^a^ |
| < 28+0 | 4 (4.3%) | 11 (8.5%) | 11 (16.7%) | <0.001^a^ | <0.001^a^ | 0.1653^a^ |
| 28+0 - 33+6 | 10 (10.8%) | 41 (31.8%) | 21 (31.8%) |  |  |  |
| > 34+0 | 79 (84.9%) | 77 (59.7%) | 34 (51.5%) |  |  |  |
| Birth weight (g) |  |  |  |  |  |  |
| <1000 | 2 (1.8%) | 10 (6%) | 17 (20.5%) | 0.004^a^ | <0.001^a^ | 0.002283^a^ |
| 1000-2000 | 20 (18.2%) | 54 (32.3%) | 24 (28.9%) |  |  |  |
| >2000 | 88 (80%) | 103 (61.7%) | 42 (50.6%) |  |  |  |
| UApH | 7.29±0.1 | 7.32±0.2 | 7.3±0.1 | 0.590^a^ | 0.443^a^ | 0.7633^a^ |
| Transfer ad NICU | 35 (31.5%) | 92 (54.1%) | 52 (62.6%) | <0.001^b^ | <0.001^b^ | 0.2495^b^ |
